# Acknowledgement to Reviewers of *Pharmaceuticals* in 2013

**DOI:** 10.3390/ph7030248

**Published:** 2014-02-27

**Authors:** Pharmaceuticals Editorial Office

**Affiliations:** MDPI AG, Klybeckstrasse 64, CH-4057 Basel, Switzerland; E-Mail: pharmaceuticals@mdpi.com

The editors of *Pharmaceuticals* would like to express their sincere gratitude to the following reviewers for assessing manuscripts in 2013:

Abreu, Paula
Adeniji, Adegoke
Albert, T.
Alevizos, Ilias
Ali, Ayad K.
Allegaert, Karel
Altmann, Friedrich
Andreu, David
Arima, Hidetoshi
Aroui, Sonia
Baker, William L.
Ballester, Pedro J.
Barashi, Itamar
Basu, Anindya
Beck, Alain
Becker, Richard C.
Bedogni, Barbara
Bendall, Linda J.
Berraondo, Pedro
Biava, Mariangela
Blum, Robert
Boisguerin, Prisca
Borissoff, Julian I.
Borroto-Escuela, Dasiel O.
Borth, Nicole
Brown, Andrew J.
Bruno, Stefano
Burke, Donald H.
Calado, Ângelo
Cemazar, Maja
Cepeda, Carlos
Chapman, Eli
Chen, Kai
Chen, Lin-Chi
Chen, Xiangmei
Cheng, Ya-Wei
Chiba, Peter
Chong, Parkson Lee-Gau
Claus, Peter
Cooke, Jonathan
Cos, Paul
Cosconati, Sandro
Cuarterolo, Miriam L.
Dardonville, Christophe
Darling, Douglas
Davies, Kay
Davis, Randall L.
Day, Andrew
De Oliveira Silva, Denise
Deber, Charles M.
Deshayes, Sébastien
Douwes, J. M.
Elferink, Lisa A.
Epstein, R. A.
Farce, Amaury
Fiaccadori, Enrico
Fischer, Gabriele
Flanagan, W Michael
Flynn, Joseph T.
Fragai, Marco
Francia, Giulio
Frasier, Iain
Froestle, Wolfgang
Fuchs, Flávio D.
Gallo, Angelo
Gambaryan, Stepan
Gebhard, Susanne
Gennaro, Lynn A.
Gilboa, Eli
Gillings, Michael
Gothe, Scott
Grudzinski, Joseph J.
Grünweller, Arnold
Gunthorpe, Martin J.
Gussio, Rick
Hagenbuch, Bruno
Hahn, Uli
Hansen, Pernille B.
Higashi, Taishi
Hiss, Jan
Holdaas, Hallvard
Howes, Melanie
Hsieh, Ching-Liang
Hsu, Yi-Chiang
Huang, Tien L.
Huang, Yue-Qiao
Huff, W. E.
Ishikawa, Sane
Ivankin, Andrey
Jackson, William
Jaeschke, Hartmut
Jalili, Ahmad
Jelokhani-Niaraki, M
Jensen, Svend
Kamangar, Faranak
Kanwar, Shailender
Karatasos, Kostas
Karlowsky, James A.
Keenan, Robert T.
Kiel, Johnathan
King, James F.
Kingston, David
Klein, Christina L.
Köhr, Georg
Komatsuzawa, Hitoshi
Kontoravdi, Cleo
Koon, Hon Wai
Kosaka, Nobuyoshi
Kota, Satya K.
Kunte, Hagen
Kwon, Y. J.
Lampkin, Stacie J.
Lappin, Terence
Lee, Myung G.
Lehrer, Robert I.
Levkau, Bodo
Lieberthal, Wilfred
Lins, L.
López-Cerero, L.
Mak, Nai K.
Mao, Hanbin
Marchandin, Hélène
Marchetti, Carla
Martin, Kathleen A.
Masters, Colin L.
Matés, José M.
Mcnamara, James
McPhee, Derek
Meszaros, Tamas
Minko, Tamara
Moore, M. D.
Moore, P. A.
Moroni, Flavio
Muncuoglu, Kosta Muncuoglu
Murdoch, Craig
Mynlieff, M.
Nagasawa, Takashi
Narváez, Manuel
Nencka, Radim
Nevárez-Moorillón, G. V.
Nielsen, Troels Tolstrup
Norata, Danilo
Okumura, Kazuhiko
Omasa, Takeshi
Oostenbrink, Chris
Orita, Masaya
Papkovsky, Dmitri B.
Park, G.W.
Partin, Kathryn M.
Perna, Annalisa
Pinteaux, Emmanuel
Piotto, Stefano P.
Pritham, Ursula A.
Prudent, R.
Raizer, Jeffrey J.
Rama, Ana Rosa
Razinkov, Vladimir I.
Reiser, Jean-Baptiste
Rodríguez, Manuel José
Rostaing, Lionel
Rudkin, Brian
Santagati, Andrea
Segar, J. L.
Serafini, G.
Shaffer, Christopher L.
Shum, Kato
Siegel, F.
Simoni, Jan
Singh, R. R.
Sitbon, Olivier
Skaper, Stephen D.
Söderhjelm, Pär
Steenholdt, Casper
Stewart, Dan L.
Stiles, Bangyan L.
Sutherland, James D.
Taft, Carlton
Tahara, Hidetoshi
Tidwell, Richard R.
Tsakris, Athanassios
Vanden Eynde, Jean Jacques
Verkhivker, Gennady M.
Vieira, Lucienne B.
Visca, Paolo
Vitetta, Luis
Wall, Nathan R.
Walton, S. Patrick
Walton, Shernaz
Wang, Chrong-Reen
Wang, Zejing
Weiergräber, M.
Weiergräber, Marco
Westermann, Bernhard
Willette, Robert N.
Xi, Jun
Xu, Y.
Yip, Wai Cheong
Zafran, Noah
Zamponi, Gerald W.
Zheng, Xiufen
Zhou, Jiehua

